# Subacute combined degeneration of the cord secondary to nitrous oxide misuse: No laughing matter

**DOI:** 10.1259/bjrcr.20200179

**Published:** 2021-02-05

**Authors:** Pia F P Charters, Hamish Duncan Morrison, Jonathan Witherick, Susan King

**Affiliations:** 1 Department of Radiology, Musgrove Park Hospital, Taunton, UK; 2 Department of Neurology, Southmead Hospital, Bristol, UK; 3 Department of Neurology, Musgrove Park Hospital, Taunton, UK

## Abstract

Nitrous oxide (N_2_O) has several traditional uses as a surgical and dental anaesthetic, as well as in aerosol spray propellants. It is the combination of analgesic and euphoric qualities coupled with accessibility as an over-the-counter household item that lends N_2_O to recreational use. Despite increasing evidence that prolonged use of N_2_O both medically and as a drug of abuse can cause disabling neurological side-effects, it remains widely used.

We present the case of an 18-year-old male who was diagnosed with subacute combined degeneration of the cord (SCDC) secondary to acute, heavy recreational use of N_2_O. The patient presented with progressive paraesthesia affecting his hands and feet associated with distal weakness. MRI of the cervical spine revealed symmetric bilateral high T2 signal within the dorsal columns extending from the level of C2 to T2 with the inverted ‘V’ sign on axial *T*_2_-weighted slices indicative of SCDC. Although vitamin B12 levels were within normal range, marked elevation of methylmalonic acid and homocysteine support the diagnosis of B12 inactivation and functional B12 deficiency, which fully resolved with treatment.

## Clinical presentation

An 18-year-old right-handed male roofer attended the emergency department with a short history of progressive sensory disturbance, distal limb weakness and gait dysfunction. He complained of pins and needles starting in his fingers and toes and spreading proximally over a two-week period in which he developed associated numbness, distal weakness and difficulty with his balance. He did not have bowel or bladder involvement.

His past medical history was unremarkable; vaccination history was complete; he was a non-smoker who took no regular medication and denied recent foreign travel or illness. He reported an alcohol intake of 20–30 units per week and habitual N_2_O inhalation from ‘whippits’ (whipped cream dispenser canisters). Two weeks prior to admission he reported inhaling over 30 whippets at a social event with symptom onset several days later.

Neurological examination was remarkable for bilateral distal vibration and proprioceptive sensory loss more pronounced in the lower limbs, mild distal limb weakness and pronounced sensory ataxia. The patella deep tendon reflexes were preserved but the ankle reflexes were absent. He had bilateral flexor plantar responses. There were no cerebellar signs, no cranial nerve abnormalities and cognitive function was normal.

## Differential diagnosis

Nutritional causes of myeloneuropathy were considered including serum copper, folic acid and vitamin E deficiencies, although vitamin B12 deficiency was suspected early due the history of N_2_O use. Guillain-Barré Syndrome was also favoured initially due to the prominent ascending peripheral neuropathy despite the lack of antecedent illness. Other aetiologies including metabolic, toxic, infective, inflammatory (transverse myelitis) and paraneoplastic disorders were also considered.

## Investigations/Imaging findings

**Figure 1.**
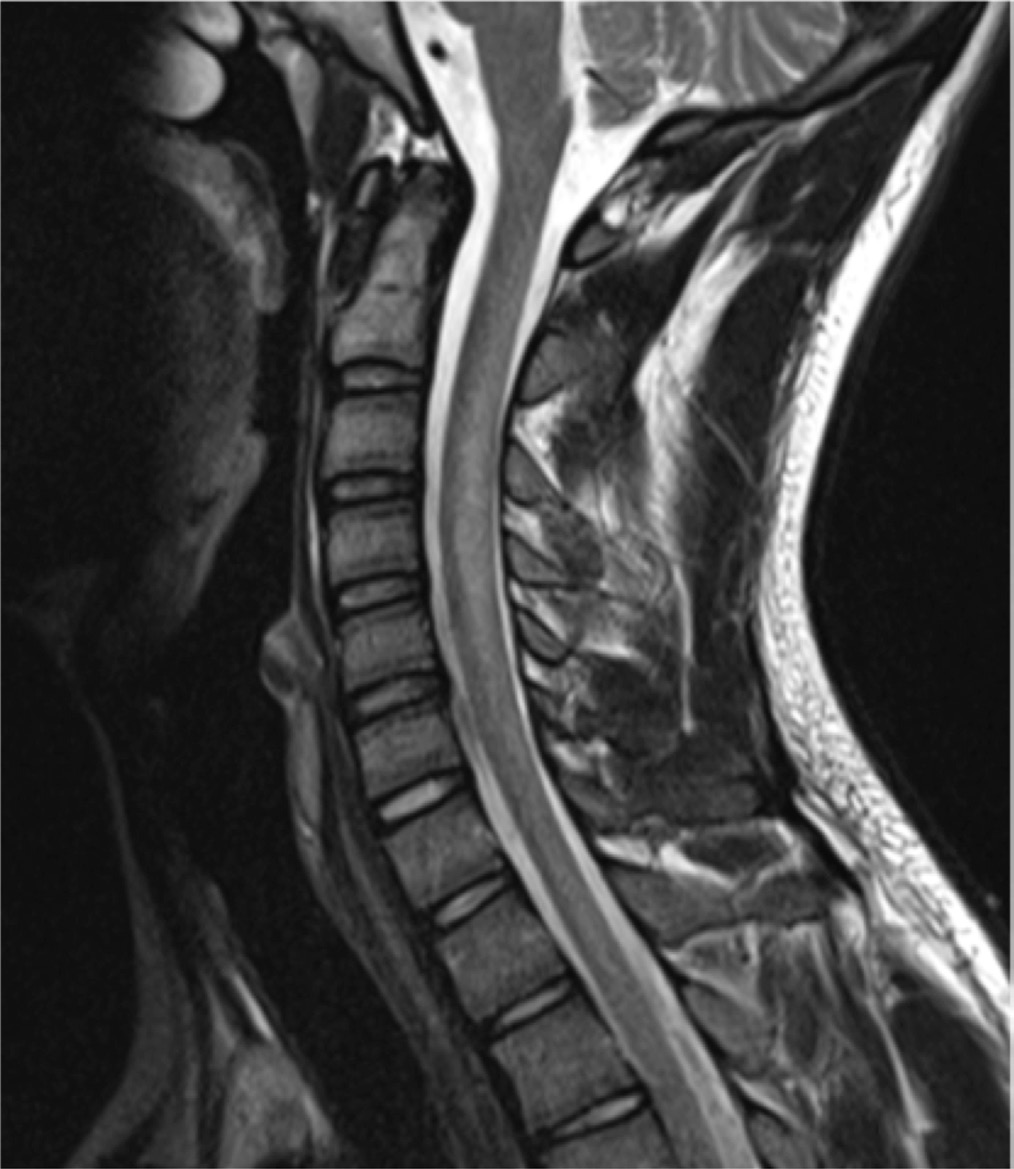
Sagittal T2 MRI cervical spine. There is high signal discretely involving the posterior column tracts with minimal cord expansion.

**Figure 2.**
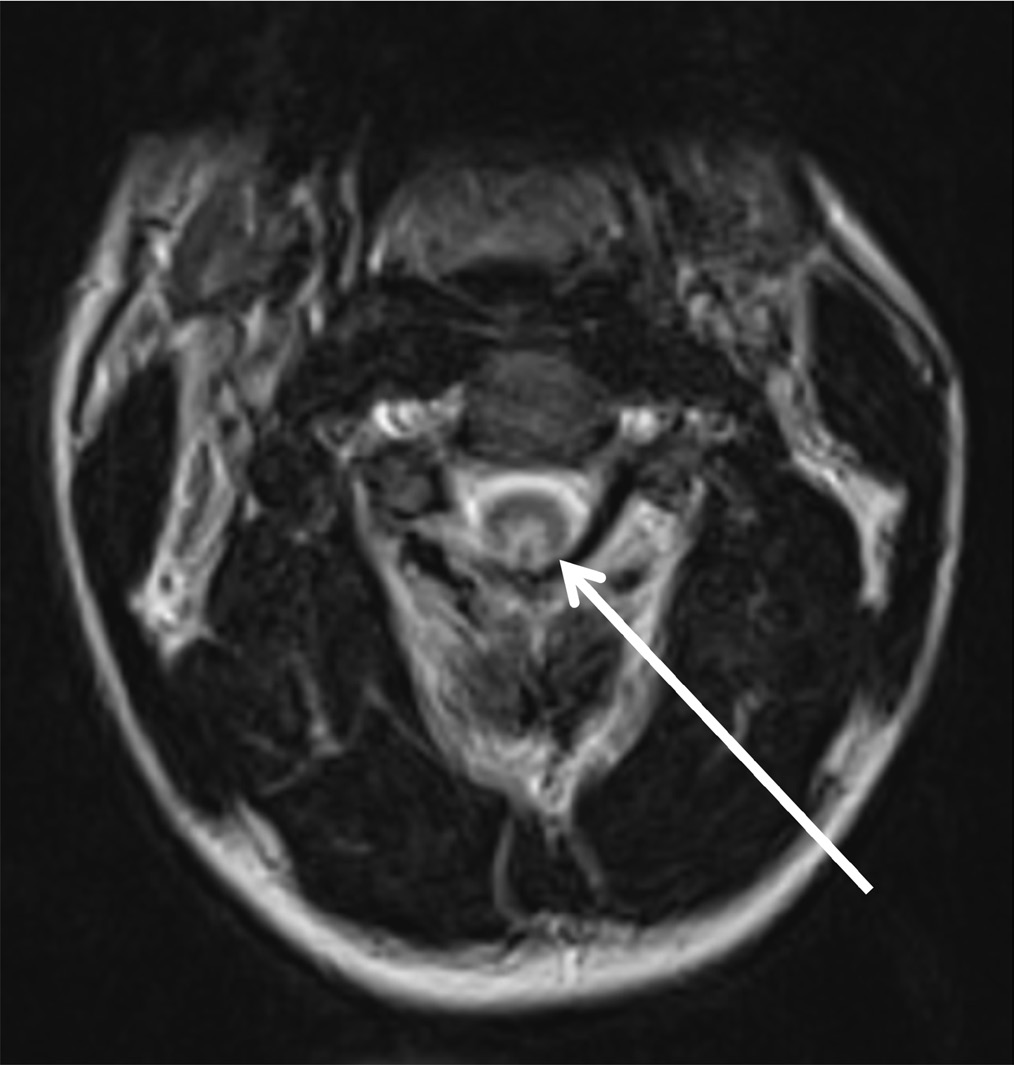
Axial T2 MRI cervical spine. Arrow: bilateral high-intensity T2 signal within the posterior funiculus, resembling the appearance of an inverted letter ‘V’.

Unenhanced MRI of the cervical spine demonstrated bilateral symmetric high T2 signal within the dorsal columns extending from the level of C2 to T2 (Figure 1), with minimal cord expansion. Bilateral high T2 signal within the posterior funiculus gave rise to the inverted ‘V’ sign,^1^ which is described in SCDC (Figure 2). Brain MRI revealed normal appearances of the parenchyma, ventricles and cerebrospinal fluid spaces.

Further workup revealed that vitamin B12 was within normal range at 171 pg ml^−1^; (145–569 pg ml^−1^). Methylmalonic acid was markedly elevated at 3.60 h µmol/L (0.10–0.42 µmol/L) as was homocysteine at 67.7 µmol/L (2.0–14.3 µmol/L) supporting the diagnosis of functional B12 deficiency. Full blood count, thyroid function, folate, copper and vitamin E levels were all normal. Antiganglioside, aquaporin four and MOG antibodies were negative. Cerebrospinal fluid analysis was entirely normal.

The combination of myelopathy, peripheral neuropathy, elevated methylmalonic acid and elevated homocysteine with typical MRI changes were overwhelmingly suggestive of SCDC secondary to functional B12 deficiency.

## Treatment

On the second day of admission, the patient commenced 1000 mcg intramuscular hydroxocobalamin injections on alternative days for 2 weeks, followed by 1000 mcg every 2 months for a year. He was discharged after 3 days of admission at which time his weakness had improved but sensory disturbance persisted.

## Outcome and follow-up

Outpatient nerve conduction (NCS) and electromyography (EMG) studies were performed following discharge. NCS showed a length-dependent, predominantly motor axonal neuropathy in the lower limbs with some slowing of sensory conduction velocities. The EMG revealed corresponding acute denervation changes in the distal leg musculature. These findings are consistent with the peripheral neuropathy seen in vitamin B12 deficiency. The clinical team did not feel that a follow-up MRI was indicated because it would not have changed management. Six months following his admission symptoms had fully resolved and he had made a complete recovery.

## Discussion

N_2_O has several traditional uses as a surgical and dental anaesthetic, as well as in aerosol spray propellants. It is this combination of analgesic and euphoric qualities coupled with accessibility as an over the-counter household item that lends N_2_O to recreational use. Despite increasing evidence that prolonged use of N_2_O both medically and as a drug of abuse can cause disabling neurological side-effects, it remains widely used.^2,3^

Since the first Global Drugs Survey in 2014, recreational N_2_O use in the UK has risen from 20 to 31%.^3^ This is reflected in the increasing number of case reports documenting vitamin B12 deficiency secondary to recreational misuse causing SCDC. A 2018 case series has emphasised the public health impact of recreational N_2_O use, postulating that many users are unaware of the harmful effect of a drug that was legal to consume prior to May 2016.^4^ Keddie and colleagues suggest future efforts focus on ascertaining the true extent of the problem, identifying at-risk groups and raising public awareness to limit long-term neurological sequelae.

SCDC is a neurodegenerative demyelinating disorder secondary to vitamin B12 deficiency. The condition is typically seen in the context of primary malnutrition, pernicious anaemia, the use of proton pump inhibitors and conditions affecting the terminal ileum such as Crohn’s disease, coeliac disease and surgical resection.^5^

**Figure 3.**
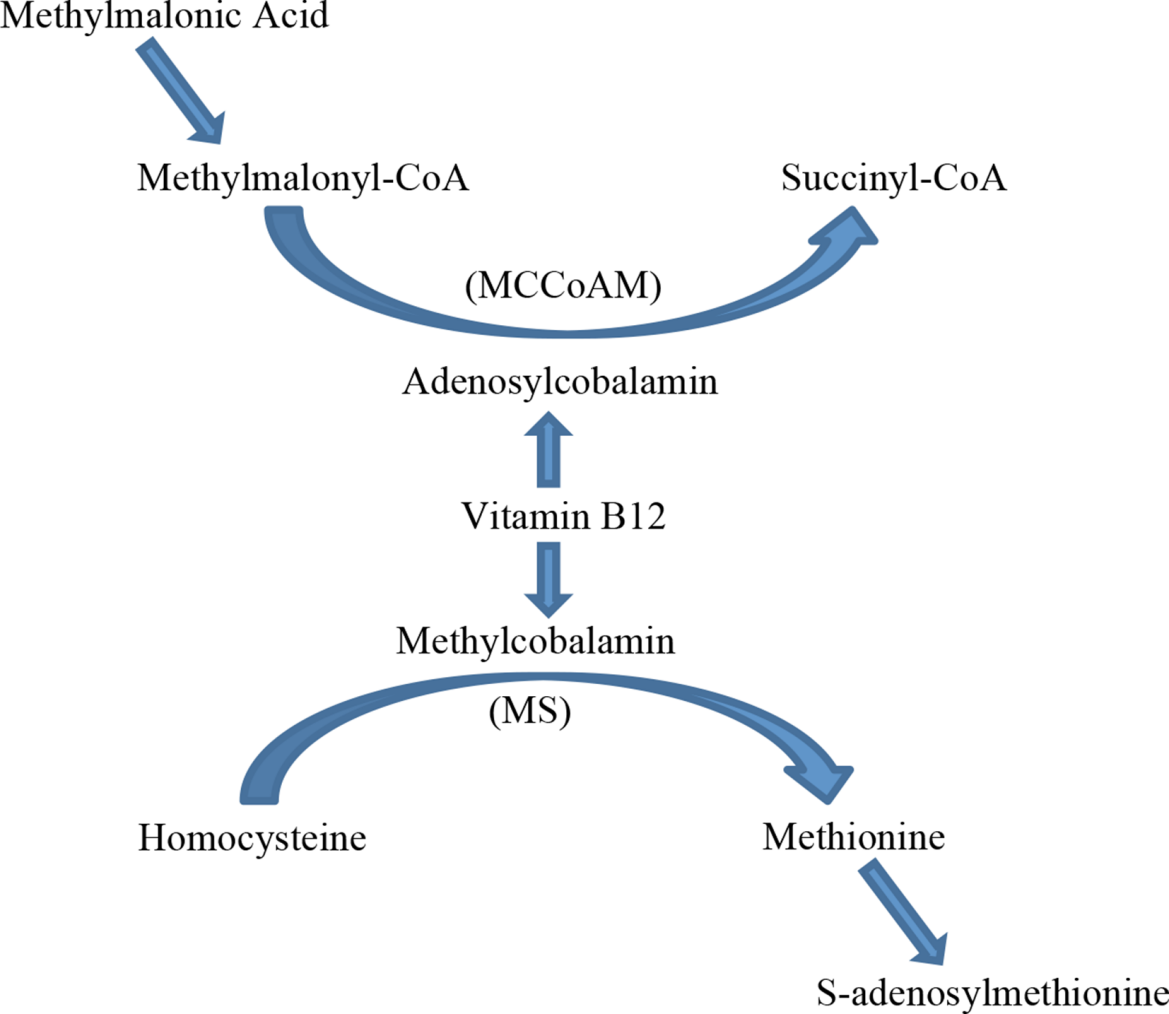
Vitamin B12 is a cofactor in conversion of methylmalonyl coenzyme A to succinyl coenzyme A and of homocysteine to methionine. Elevated levels of the substrates methylmalonic acid and homocysteine can be used to identify vitamin B12 inactivation despite normal serum B12 concentrations. MS = methionine synthase, MCCoAM = methylmalonyl coenzyme CoA mutase.

Active vitamin B12 is essential for several enzyme-catalysed reactions, most notably in the synthesis of myelin.^6^ Active vitamin B12 comprises of methylcobalamin and adenosylcobalamin coenzymes, methylcobalamin is a cofactor in the synthesis of methionine and adenosylcobalamin is a cofactor in the synthesis of succinyl Cofactor A (CoA). Methylcobalamin methylates homocysteine by acting as a cofactor for the enzyme methionine synthase. Methionine synthase is a precursor to methionine and ultimately S-adenosylmethionine^6^ (Figure 3). S-adenosylmethionine is the major methyl donor required for numerous biochemical functions and cellular processes including the synthesis of nucleic acids, catecholamines, and myelin sheath phospholipids.^7^

Adenosylcobalamin, the second active form of vitamin B12, acts as a cofactor for the enzyme methylmalonyl coenzyme CoA mutase which converts methylmalonyl-CoA into succinyl-CoA (Figure 3). Vitamin B12 deficiency leads to accumulation of methylmalonyl-CoA, high levels of which are used instead of malonyl-CoA for fatty acid synthesis. Myelin synthesised with abnormal methylmalonyl-CoA fatty acids is unstable and prone to degradation, thus causing demyelinating neuropathy.^7^

N_2_O inactivates vitamin B12 (cobalamin) by irreversibly oxidising the cobalt ion.^8^ Inactive vitamin B12 equates to a functional deficiency of methylcobalamin and adenosylcobalamin resulting in elevated levels of both methylmalonic acid and homocysteine, as seen in our patient.^4^ Several articles have been published reporting myeloneuropathy in patients exposed to N_2_O in spite of having normal vitamin B12 levels, levels may be normal in up to 30% of patients.^9,10^ If demyelination is suspected then is important to look for elevated levels of the B12-dependent metabolites, methylmalonic acid and homocysteine, despite normal serum vitamin B12 levels.^9^

Loss of normal myelin around axons in the central nervous system manifests as high signal in the white matter on *T*_2_-weighted MRI.^7–10^ The lesions are typically non-enhancing and begin in the upper thoracic cord. Progression may be ascending or descending. Demyelination in SCDC has a predilection for the dorsal columns, it unclear why the corticospinal white matter tracts are not involved.^8^ There are rarer reports of anterior column involvement.^11^ Bilateral high T2 signal within the posterior funiculus gives rise to the inverted ‘V’ sign or inverted “rabbit ears” appearance on axial imaging.^1^ Axial T2 images are more sensitive at detecting cord lesions as faint signal abnormalities which are subject to partial voluming on sagittal sequences.^7^ Demyelination of the thoracic dorsal columns appears to be the earliest manifestation of vitamin B12 deficiency and is considered a reversible pathological change in SCDC. Other less frequently documented MRI findings include minimal spinal cord enlargement, dorsal spinal cord hypointensity on *T_1_* weighted imaging, T2 hyperintense signal in lateral columns of cord and cerebral white matter.^1,7^ Imaging differentials include infectious and post-infectious myelitis, metabolic and nutritional disturbance (including copper and vitamin E deficiency), neoplasia and paraneoplastic myelopathy, radiation myelitis, traumatic cord injury, multiple sclerosis and neuromyelitis optica spectrum disorder.

Not all patients will present with typical MRI findings and it is notable that investigations are complementary and not exclusive. There are reports of patients with normal cord imaging and no clinical evidence of myeloneuropathy who were found to have sensorimotor neuropathy with demyelinating features on neurophysiology.^12^ In our case, Guillain-Barré Syndrome was suspected before neuroimaging and later biochemical screening revealed a functional B12 deficiency. There are reports of patients with SCDC going on to receive intravenous immunoglobulin therapy due to initial suspicion of Guillain–Barré Syndrome.^12^

Management of SCDC secondary to B12 inactivation includes cessation of N_2_O inhalation and high-dose intramuscular hydroxocobalamin for 12 months as described above.^5,13^ Not all patients achieve full neurological recovery.^7–9,12^

## Learning points

- Diagnosing subacute combined degeneration of the cord secondary to N_2_O misuse requires a thorough medical and social history, clinical examination demonstrating myelopathy and/or peripheral neuropathy and/or MRI findings consistent with a typical demyelination pattern.
- Vitamin B12 deficiency cannot be excluded by serum B12 levels alone and clinical suspicion should prompt testing for functional B12 deficiency.
- Functional B12 deficiency is biochemically supported by elevated homocysteine and methylmalonic acid and can respond favourably to treatment.
- Demyelination classically occurs in the cervical or upper thoracic cord. Axial MRI demonstrates high T2 signal within the dorsal columns giving rise to the inverted ‘V’ sign. Sagittal MRI typically demonstrates dorsal spinal cord high T2 signal.
